# Novel CD37, Humanized CD37 and Bi-Specific Humanized CD37-CD19 CAR-T Cells Specifically Target Lymphoma

**DOI:** 10.3390/cancers13050981

**Published:** 2021-02-26

**Authors:** Vita Golubovskaya, Hua Zhou, Feng Li, Michael Valentine, Jinying Sun, Robert Berahovich, Shirley Xu, Milton Quintanilla, Man Cheong Ma, John Sienkiewicz, Yanwei Huang, Lijun Wu

**Affiliations:** 1 Promab Biotechnologies, 2600 Hilltop Drive, Richmond, CA 94806, USA; hua.zhou@promab.com (H.Z.); feng.li@promab.com (F.L.); michael.valentine@promab.com (M.V.); sunnie.sun@promab.com (J.S.); robert.berahovich@promab.com (R.B.); shirley.xu@promab.com (S.X.); milton.quintanilla@promab.com (M.Q.); iris.ma@promab.com (M.C.M.); john.sienkiewicz@promab.com (J.S.); yanwei.huang@promab.com (Y.H.); 2 Biology and Environmental Science College, Hunan University of Arts and Science, Changde 415000, China; 3 Forevertek Biotechnology, Janshan Road, Changsha Hi-Tech Industrial Development Zone, Changsha 410205, China

**Keywords:** chimeric antigen receptor, CAR-T cells, CD37, CD19, immunotherapy, cell therapy, tumor antigen, lymphoma

## Abstract

**Simple Summary:**

Chimeric antigen receptor (CAR) T cell therapy represents a major advancement in cancer treatment. Recently, FDA approved CAR-T cells directed against the CD19 protein for treatment of leukemia and lymphoma. In spite of impressive clinical responses with CD19-CAR-T cells, some patients demonstrate disease relapse due to either antigen loss, cancer heterogeneity or other mechanisms. Novel CAR-T cells and targets are important for the field. This report describes novel CD37, humanized CD37 and bispecific humanized CD37-CD19-CAR-T cells targeting both CD37 and CD19. The study demonstrates that these novel CAR-T cells specifically targeted either CD37 positive or CD37 and CD19-positive cells with endogenous and exogenous protein expression and provides a basis for future clinical studies.

**Abstract:**

CD19 and CD37 proteins are highly expressed in B-cell lymphoma and have been successfully targeted with different monotherapies, including chimeric antigen receptor (CAR)-T cell therapy. The goal of this study was to target lymphoma with novel CD37, humanized CD37, and bi-specific humanized CD37-CD19 CAR-T cells. A novel mouse monoclonal anti-human CD37 antibody (clone 2B8D12F2D4) was generated with high binding affinity for CD37 antigen (KD = 1.6 nM). The CD37 antibody specifically recognized cell surface CD37 protein in lymphoma cells and not in multiple myeloma or other types of cancer. The mouse and humanized CD37-CAR-T cells specifically killed Raji and CHO-CD37 cells and secreted IFN-gamma. In addition, we generated bi-specific humanized hCD37-CD19 CAR-T cells that specifically killed Raji cells, CHO-CD37, and Hela-CD19 cells and did not kill control CHO or Hela cells. Moreover, the hCD37-CD19 CAR-T cells secreted IFN-gamma against CD37-positive and CD19-positive target CHO-CD37, Hela-CD19 cells, respectively, but not against CD19 and CD37-negative parental cell line. The bi-specific hCD37-CD19 significantly inhibited Raji xenograft tumor growth and prolonged mouse survival in NOD scid gamma mouse (NSG) mouse model. This study demonstrates that novel humanized CD37 and humanized CD37-CD19 CAR-T cells specifically targeted either CD37 positive or CD37 and CD19-positive cells and provides a basis for future clinical studies.

## 1. Introduction

Chimeric antigen receptor (CAR) T cell therapy is an exciting and novel area of immuno-oncology research [1,2,3]. CAR-T cells have been tested against several targets for hematological cancers, such as CD19, CD20, CD22, CD123, BCMA, and others in clinical trials [4,5,6,7,8,9,10,11,12,13]. Novel approaches and targets are being developed to overcome challenges to existing cell therapies, such as loss of antigen, an immunosuppressive tumor microenvironment, and limited persistence of CAR-T cells [4,12,14,15,16,17]. Recently, novel anti-CD37 CAR-T cell therapy was developed for lymphoma patients [18,19].

CD37 is highly expressed in many hematological cancers, such as non-Hodgkin’s lymphoma (NHL), diffuse large B-cell lymphoma (DLBCL), chronic lymphocytic leukemia (CLL), acute lymphocytic leukemia (ALL), and in some peripheral and cutaneous T cell lymphomas [20,21,22], and absent or weakly expressed in multiple myeloma and Hodgkin’s lymphoma [23]. CD37 is a 40–52 kDa heavily glycosylated member of the transmembrane 4 superfamily (TM4SF) of tetraspanin proteins [24,25]. CD37 plays a role in integrin, AKT, PI3-Kinase-dependent survival, and apoptotic signaling, motility, immune response signaling via activation of dendritic cell migration [25,26].

CD37 expressing cancers have been targeted with several antibody-based therapies, including Fc engineered antibodies (BI836826), drug or radio immunoconjugates (maytansinoid DM1 IMGN529; monomethyl auristatin E, AGS67E, and (^177^ Lu) Betalutin), DuoHexaBody-CD37, and single-chain variable fragments (ScFv) (Otlertuzumab/TRU-016), either alone or in combination with rituximab, chemotherapy, or other agents [22,27,28,29,30,31,32].

Recently, FDA-approved CD19-CAR-T cells (Kymriah (tisagenlecleucel) and Yescarta (axicabtagene ciloleucel) have successfully treated patients with CD19+ B-cell leukemias [8,33]. However, the relapse due to loss of the CD19 antigen via alternative splicing or mutations leading to loss of the protein transmembrane domain has been observed [34,35]. To improve the efficacy of CAR-T cells in case of loss of antigen, dual, tandem, or bispecific CAR-T cells were generated which target two different antigens, such as CD19/CD20 [36,37]; CD19/CD22 [38,39]; CD19/CD123 [40].

This report demonstrates the efficacy of three novel CAR-T cells derived from CD37 antibody, clone 2B8D12F2D4: mouse CD37, humanized hCD37 CAR-T cells, and bispecific hCD37-CD19 CAR-T cells against lymphoma. Data show effective and specific targeting of lymphoma cells expressing CD37 in vitro, and decreased tumor burden, and increased median survival in a xenograft model in vivo, providing a solid basis for future clinical studies.

## 2. Results

### 2.1. CD37 Antibody Clone 2B8D12F2D4 Binds Specifically and Selectively with High Affinity to CD37 Antigen

**Figure 1:**
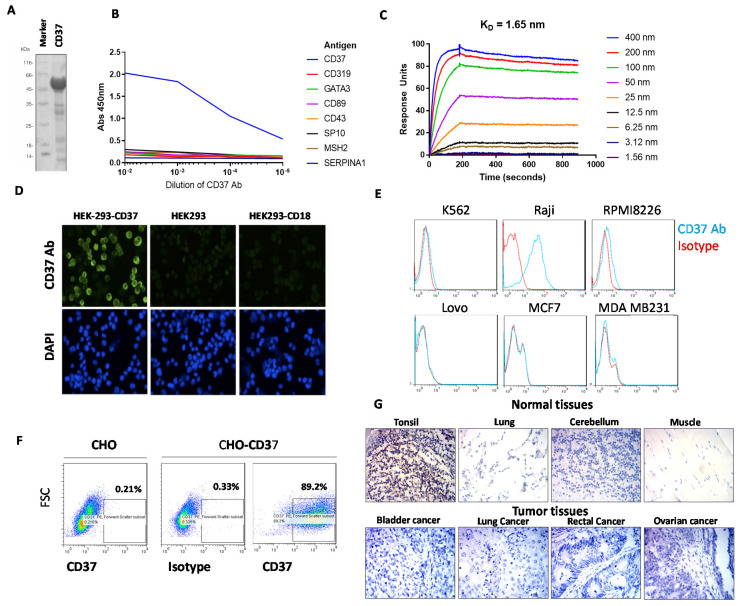
(**A**). Recombinant CD37 extracellular domain protein used for enzyme-linked immunosorbent assay, ELISA assay. The CD37 protein had C-terminal Maltose Binding Protein, MBP and His tag. SDS (sodium dodecyl sulfate) gel shows 56 kDa CD37 protein. (**B**). ELISA shows binding of CD37 antibody 2B8D12F2D4 (2B8) clone to CD37 protein and no binding to other unrelated control proteins. (**C**). Surface plasmon resonance kinetic data collected on Biacore with anti-CD37 2B8 antibody bound to mouse capture chip and titration of CD37-His. KD of 1.65 nm was measured from two independent experiments. (**D**). Immunostaining shows binding of CD37 2B8 clone antibody to CD37 antigen in HEK293-CD37 cells but not in HEK293-CD18 and HEK293 cells. Indirect immunofluorescence microscopy was performed with anti-CD37 2B8 followed by Goat-Anti-mouse IgG Alexa 488 (top row) and counterstained with DAPI (4′,6-diamidino-2-phenylindole) nuclear stain (bottom row). (**E**). FACS shows positive staining of CD37 2B8 antibody in CD37-positive Raji cells but not in other cancer cell lines. (**F**). FACS with CD37 antibody shows specific detection of cell surface CD37 in CHO-CD37 cells but not in CHO cells. (**G**). Indirect immunohistochemistry was performed on health (upper panel) and tumor (lower panel) adult tissue sections with anti-CD37 2B8 staining, followed by anti-mouse HRP (horseradish peroxidase). Positive staining was observed in tonsil, a lymphoid tissue, but not in normal lung, cerebellum, or muscle. Negative staining was observed in representative tumor samples.

Several murine anti-human CD37 mAbs were isolated from hybridoma and screened for binding to recombinant human CD37-Maltose binding protein (MBP)-His antigen (Figure 1A) and seven other unrelated proteins (Figure 1B). CD37 antibody, clone 2B8D12F2D4, hereafter referred to as 2B8, specifically bound to CD37 antigen and did not bind to any of the other proteins tested. (Figure 1B). To detect the affinity of the CD37 antibody, a kinetic surface plasmon resonance experiment was performed on a Biacore with CD37-His protein. The CD37 antibody bound to CD37 antigen with high affinity, with binding constant KD of 1.65 nM (Figure 1C).

To detect binding of CD37 antibody on the cell surface, we transfected human embryonic kidney, HEK-293 cells either with CD37 antigen plasmid or with negative control CD18 plasmid and showed specific binding of CD37 antibody 2B8 clone to CD37 in HEK-293-CD37 cells but not in control HEK293-CD18 or HEK293 cells (Figure 1D). In addition, Fluorescence Activated Cell Sorting, FACS analysis with Raji lymphoma cells demonstrated positive staining with CD37 antibody but not with other K562 leukemia cells or multiple myeloma RPMI8226, colon cancer Lovo cells, breast cancer MCF-7, or MDA-231 cells (Figure 1E). In addition, the CD37 antibody detected CD37 antigen in three primary leukemia samples (Supplementary Figure S1). This shows that the CD37 antibody specifically binds CD37 in lymphoma cells with endogenous expression of CD37 but not in other types of cancer. To additionally test the specificity of the CD37 antibody, we tested CHO-CD37 and CHO cells (Figure 1F).

We show that CD37 antibody has negative FACS staining in CHO cells but high staining in CHO-CD37 cells (Figure 1F). There was also negative staining with IgG1 isotype control in CHO-CD37 cells (Figure 1F). These and the above data demonstrate 2B8 bound to surface-expressed CD37 and not to other surface proteins.

Immunohistochemical staining (IHC) demonstrated low or negative staining in many normal tissues (esophagus, stomach, rectum, thyroid, kidney, lung, muscle, brain) (Supplementary Table S1) but increased staining in tonsils where lymphocytes were present (Figure 1G, upper panel). There was also negative staining in most types of cancer tumors (ovarian, lung, cervical, bladder, lung, prostate, rectal, gastric cancer) (Figure S1) (Figure 1G, lower panel).

Thus, the specific binding of CD37 to extracellular CD37 antigen in lymphoma cells makes this novel antibody suitable for CAR generation.

### 2.2. CD37-CAR-T Cells Specifically Target CD37-Positive Cells

**Figure 2:**
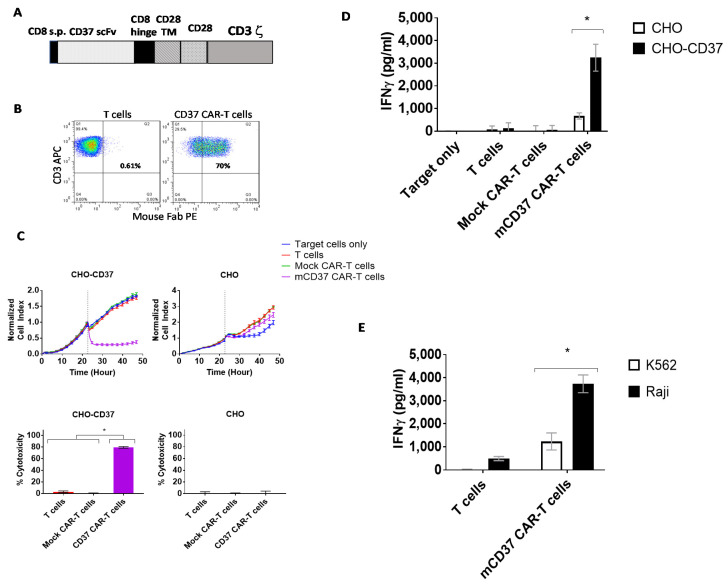
The specific CD37-chimeric antigen receptor (CAR)-T cell activity against CD37-positive cells in vitro. (**A**). The structure of CD37-CAR. The mouse ScFv (single-chain variable fragment) was used with CD8 hinge, CD28 transmembrane/costimulatory domains, and CD3 zeta activation domain. CD8 s.p-CD8 alpha signaling peptide; TM-transmembrane. (**B**). FACS with mouse F(ab)2 antibody (mFAB) detected CAR-positive cells. (**C**). Real-time cytotoxicity assay (RTCA) showed specific killing activity of CD37-CAR-T cells against CHO-CD37 cells but not CHO cells (upper panels). Lower panels: Percent cytotoxicity calculated at the end of the experiment. Significantly high cytotoxicity was observed against CHO-CD37 for CD37-CAR-T cells. *p* < 0.0001, One-Way ANOVA followed by Sidak multiple comparisons test. (**D**). Interferon-gamma (IFN-γ) secretion by CD37-CAR-T cells against CHO-CD37 cells is significantly higher than against CHO cells. asterisk *, *p* < 0.0001, two-way ANOVA *p* <0.0001, followed by Tukey’s multiple comparison test. (**E**). Secretion of IFN-gamma by CD37-CAR-T cells is significantly higher with Raji cells than with CD37-negative K562 cells. asterisk *, *p* < 0.005, mCD37 CAR-T cells with Raji cells versus same CAR-T cells with K562 cells, Student’s *t*-test.

We generated CAR with CD37 2B8 ScFv with a CD28 costimulatory domain and CD3 zeta activation domain (Figure 2A). The CD37-CAR-T cells were >70% CAR-positive after transduction with CD37-CAR lentivirus (Figure 2B). Then CD37-CAR-T cells were tested in Real-time cytotoxicity assay (RTCA) using target CD37-positive CHO-CD37 and CD37-negative CHO cells. CD37-CAR-T cells killed CHO-CD37 cells but did not kill CHO cells (Figure 2C, upper panels). Cytotoxicity was significantly higher for CD37 CAR-T cells than T cells or mock CAR-T cells (Figure 2C, bottom panels). IFN-γ released by CD37 CAR-T cells in response to CHO-CD37 target cells was significantly higher than in response to CHO cells (Figure 2D). Significantly higher secreted levels of IFN-gamma by CD37-CAR-T cells were detected with CD37-positive Raji cells than with CD37-negative K562 cells (Figure 2E). Thus, novel CD37 2B8 ScFv-CAR-T cells are effective and specific against CD37-positive target cells with exogenous and endogenous expression of CD37.

### 2.3. Humanized CD37-CAR-T Cells Specifically Target CD37-Positive Cells

**Figure 3:**
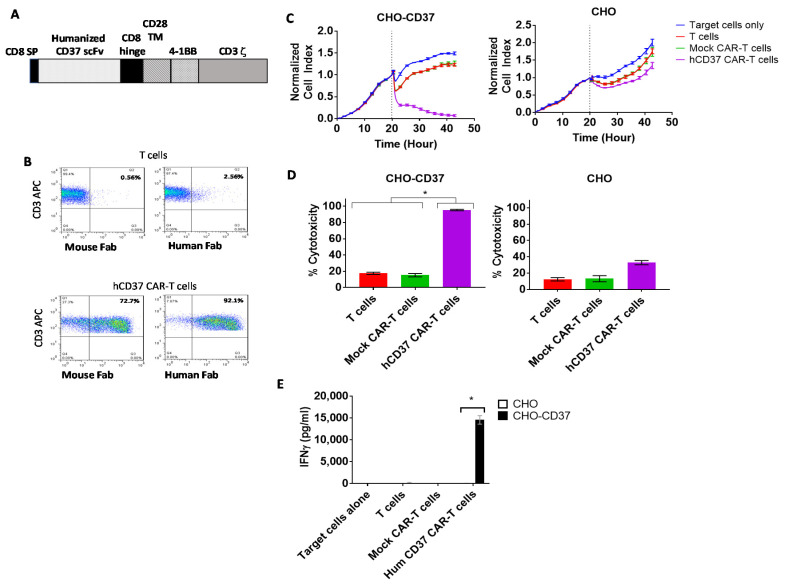
The humanized CD37-CAR-T cells specifically target CD37-positive cells. (**A**). The structure of humanized CD37-CAR-T cells. The structure includes CD8 signaling peptide; humanized CD37 ScFv, CD8 hinge, CD28 TM (transmembrane domain); 41BB domain; CD3 zeta activation domain. (**B**). FACS with mouse and human FAB detected CAR-positive cells. (**C**). Humanized CD37-CAR-T cells killed CHO-CD37-positive cells and did not kill CHO cells. (**D**). Quantification of cytotoxicity shows significantly higher killing by CD37CAR-T cells in CHO-CD37 cells than Mock and T cells. *, hCD37 CAR-T cells with CHO-CD37 cells versus T and Mock CAR-T cells, *p* < 0.0001, One-Way ANOVA followed by Dunnett’s Multiple Comparison Test. (**E**). hCD37-CAR-T cells secrete significantly higher IFN-gamma with CHO-CD37 cells than with CHO cells. *, *p* < 0.05, IFN-gamma of humanized Hum CD37 CAR-T cells with CHO-CD37 cells versus same CAR-T cells with CHO cells by Student’s *t*-test.

We humanized CD37 VH and VL, as described in Materials and Methods, and generated lentiviral humanized CD37 CAR with a 4-1BB costimulatory domain and CD3 activation domain, called hCD37 CAR (Figure 3A). Surface expression of the CAR was detected by FACS with both anti-mouse Fab (72% positive) and anti-Human Fab (92% positive) (Figure 3B). In real-time cytotoxicity assay against CHO-CD37 and CHO cells, humanized anti-CD37 CAR-T cells effectively killed CHO-CD37 cells and demonstrated limited or no killing of CHO cells (Figure 3C). Cytotoxicity of humanized CD37 CAR-T cells against CHO-CD37 (95.3% ± 0.8%) was significantly higher than non-transduced T cells (17.5% ± 1.3%) or mock CAR-T cells (Figure 3D). The hCD37-CAR-T cells secreted significantly higher levels of IFN-gamma with CD37-positive target cells than with CD37-negative cells (Figure 3E). Thus, humanized CD37-CAR-T cells specifically target CD37-positive cells.

### 2.4. Bispecific Humanized CD37-CD19 CAR-T Cells Specifically Target CD37-Positive Cells

**Figure 4:**
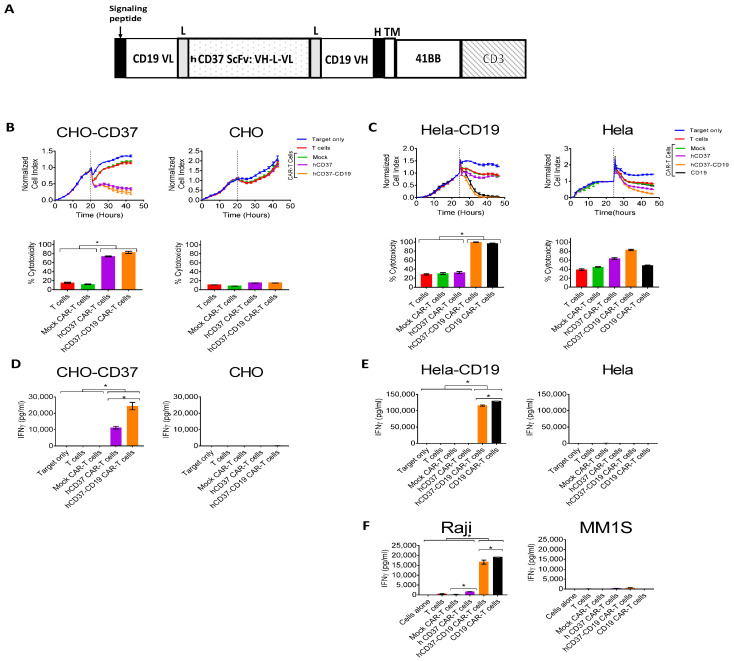
Bi-specific hCD37-CD19-CAR-T cells specifically target CD37-positive and CD19-positive cells. (**A**). The structure of bi-specific CD37-CD19 CAR-T cells. VL-light chain; VH-heavy chain; ScFv-single chain variable fragment; L-linker; H-hinge; TM, transmembrane domain. (**B**). RTCA activity of hCD37-CD19-CAR-T cells with CHO-CD37 cells (left) and CHO cells (right). Cytotoxicity of bispecific CD37-CD19 CAR-T cells against CHO-CD37 cells was significantly higher than that of humanized CD37 CAR-T cells, *, *p* < 0.0001, hCD37 and hCD37-CD19-CAR-T cells with CHO-CD37 cells vs T and Mock CAR-T cells, One-Way ANOVA followed by Sidak’s multiple comparison test *p* = 0.0006. (**C**). RTCA activity of hCD37-CD19-CAR-T cells with Hela-CD19 cells (left) and Hela cells (right). Quantification of RTCA at the end time point is shown under the RTCA plots. * *p* < 0.0001, * hCD37-CD19 CAR-T cells and CD19 CAR-T cells with Hela-CD19 cells vs T cells, Mock CAR-T cells, CD37 CAR-T cells by One-Way ANOVA followed by Sidak’s multiple comparison as in B. (**D**). IFN-gamma secretion by hCD37-CD19-CAR-T cells was significantly higher with CHO-CD37 cells than with CHO cells. * *p* < 0.0001, CD37, hCD37-CD19 CAR-T cells vs other groups with CHO-CD37 cells by One-way ANOVA followed by Tukey’s test. (**E**). IFN-gamma secretion by CD37-CAR-T cells was significantly higher with Hela-CD19 cells than with Hela cells, * *p* < 0.05, hCD37-CD19 and CD19 CAR-T cells with Hela-CD19 cells vs other groups with Raji cells, Student’s *t*-test. (**F**). IFN gamma secretion by hCD37-CD19-CAR-T cells against Raji cells was significantly higher than with CD37-negative multiple myeloma MM1S cells, *p* < 0.001, * hCD37, hCD37-CD19 and CD19 CAR-T cells with Raji cells vs Mock CAR-T cell groups with Raji cells by Tukey’s test.

Next, we tested the efficacy of bi-specific humanized hCD37-CD19 CAR-T cells in vitro. To generate bi-specific humanized CD37-CD19 CAR-T cells, we used the following design as shown in Figure 4A with humanized CD37 ScFv and mouse CD19 FM63 ScFv [41]. These CAR-T cells had a surface expression of CAR as detected by FACS with anti-mouse and anti-human Fab antibodies (not shown). Real-time cytotoxicity assays were performed against CHO-CD37 and CHO cells (Figure 4B) and against Hela-CD19 and Hela cells (Figure 4C). Killing by bispecific hCD37-CD19 CAR-T cells was compared to CAR-T cells expressing monospecific hCD37 CAR or CD19 CAR. Bi-specific hCD37-CD19 CAR-T cells killed CHO-CD37 as effective as single hCD37-CAR-T cells and did not kill CHO cells (Figure 4B).

The hCD37-CD19 CAR-T cells also killed Hela-CD19 target cells and did not kill Hela cells (Figure 4C). As expected, single hCD37-CAR-T cells did not kill Hela-CD19 cells. The hCD37-CD19 CAR-T cells and hCD37-CAR-T cells secreted significantly higher levels of IFN-gamma against CHO-CD37 cells versus CHO cells (Figure 4D). Both hCD37-CD19 and CD19-CAR-T cells secreted significantly higher levels of IFN-gamma against Hela-CD19 target cells but not against Hela cells (Figure 4E).

In separate coculture experiments, IFN-γ release against Raji cells or MM1s cells was measured (Figure 4F). Both CD37-CD19 CAR-T cells and CD19 CAR-T cells had significantly more IFN-γ release than humanized CD37 CAR-T cells, mock CAR-T cells, and non-transduced T cells (*p* < 0.0001, Tukey’s test) (Figure 4F). The secretion of IFN-gamma was significantly higher for CD37-CD19-CAR-T cells against Raji cells than against MM1S cells.

Thus, hCD37-CD19 CAR-T cells demonstrate high and specific efficacy against CD37 and CD19-positive target cells in vitro.

### 2.5. Humanized CD37-CD19 CAR-T Cells Inhibit Raji Lymphoma Xenograft Tumor Growth and Prolong Mice Survival

At first, we tested the efficacy of CD37-CAR-T cells in vivo and performed survival analysis using a Raji-xenograft tumor model after an injection of mouse CD37-CAR-T cells and humanized CD37-CAR-T cells (Figure S2). Mouse and humanized CD37-CAR-T cells prolonged mouse survival as well as CD19-CAR-T cells (Supplementary Figure S2).

**Figure 5:**
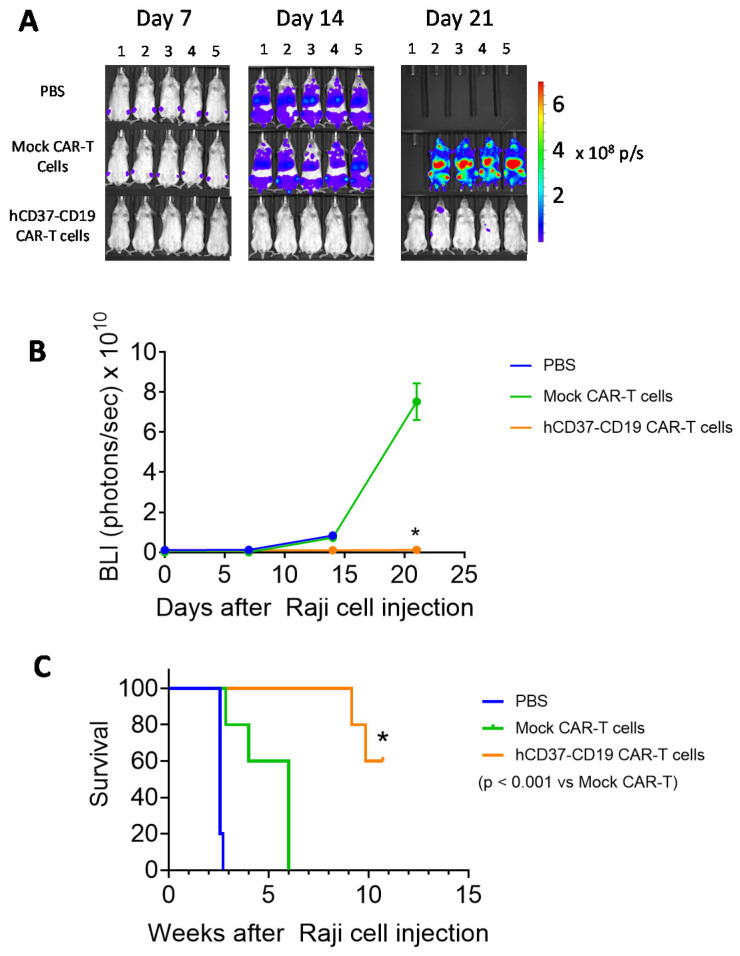
hCD37-CD19-CAR-T cells significantly block Raji xenograft tumor growth in vivo. (**A**). In vivo imaging of Raji tumors in mice on days 7, 14, and 21 following Raji-Luc+ cells injection with the vehicle, mock-CAR-T cells, or bispecific CD37-CD19 CAR-T cell-treated groups (*n* = 5 each). (**B**). Tumor luminescence flux from In Vivo Imaging System, IVIS imaging. Vehicle-treated mice had died by day 14. * *p* < 0.05, hCD37-CD19 CAR-T cells vs. Mock CAR-T cells, Student’s *t*-test. (**C**). hCD37-CD19-CAR-T cells significantly prolong mouse survival in the Raji xenograft model. Kaplan–Myer curve is shown, *p* < 0.05, log–rank test hCD37-CD19 CAR-T cell-treated vs. Mock CAR-T cell-treated group.

To test the efficacy of the bispecific humanized CD37-CD19 CAR-T cells in vivo, Nod Scid Gamma, NSG mice were injected with 5 × 10^5^ Raji-Luc cells followed 24 h later with 1 × 10^7^ humanized CD37-CD19 CAR-T cells, mock CAR-T cells, or vehicle. Tumor luminescence was detected in mice treated with mock CAR-T cells or vehicle but not in mice treated with CD37-CD19 CAR-T cells (Figure 5A). Tumor luminescence in CD37-CD19 CAR-T cell treated mice was significantly lower than in mock CAR-T cell treated mice (Figure 5B). Survival of CD37-CD19 CAR-T cell treated group was significantly longer (≥75 days) (log–rank test *p* < 0.0001) than vehicle (18 days) and mock CAR-T cell treated groups (Figure 5C). Thus, humanized CD37 CAR-T cells and bi-specific hCD37-CD19 CAR-T cells are efficacious in the model in vivo.

## 3. Discussion

The present report demonstrates the efficacy of novel CD37-CAR-T cells and bispecific hCD37-CD19 CAR-T cells in vitro and in vivo. The novel CD37 antibody clone 2B8 was specific for the CD37 extracellular domain and bound with high affinity.

Standard of care for Non-Hodgkin lymphoma may include chemotherapy combined with anti-CD20 Ab (Rituximab) [21]. Relapse frequently occurs, demanding novel approaches. CD37 has been identified as a possible target for NHL immunotherapy. Anti-CD37-radioimmunoconjugates [27], duaHexabody CD37R0 [32], CD37 chimeric antibody (BI 836826) [29], and recently, CAR-T cells [18,19] have been tested.

The CD37-CAR-T cell therapy is especially important during lymphoma relapse when CD19 antigen is lost in lymphoma by either alternative splicing or other mechanisms, such as mutations [18]. Thus, CD37-CAR-T cells can improve the outcome of CD19-negative relapsed lymphoma patients. Bi-specific CD37-CD19 CAR-T cells can be important to increase the efficacy of CD19-CAR-T cells and also important in case of CD37 antigen loss due to missense mutations or other mechanisms [42].

The CD19-CD37 CAR-T cells were described by [19], but they had a different structure than described in this report. The CD37-CD19 CAR design presented here is similar to the CD19-CD22 CAR design described by [39]. In the future, clinical studies will show the advantages of each CAR. In addition, this study shows that humanized CD37-CD19 CAR-T cells effectively blocked lymphoma growth in vivo that can be advantageous in case of downregulation of either CD19 or CD37 pathways or for more efficient targeting of both antigens. Moreover, humanized CD37 ScFv can also be used for the development of other approaches, such as antibody conjugates or bispecific antibodies.

Interestingly, IFN-gamma secreted by CD19-CAR-T cells was higher than by hCD37-CAR-T cells. The differences by CAR-T cells in the secretion of IFN-gamma can be explained by 3D conformation of antigen, distance to the membrane of the antibody epitope, and other mechanisms. The lower secretion of IFN-gamma by CD37-CAR-T cells can be important for potentially reducing of cytokine release storm (CRS) in the clinic.

Since lymphoma tumors are heterogeneous and surrounded by a microenvironment that can block immune response functions [43], the combination therapy of CAR-T cells with checkpoint inhibitors, checkpoint blocking antibodies with agonist antibodies inducing an immune response, or with small molecules can overcome these barriers. Tumor-associated macrophages (TAM)s were also reported to block immune responses in leukemia and lymphoma, and novel therapies needed to repress TAM in combination with targeting lymphoma cells [44]. Thus, future combination therapies can be tested in preclinical and clinical studies that target both the tumor and tumor microenvironment.

The novel CD37, humanized CD37, and CD37-CD19 CAR-T cells provide a basis for future clinical studies.

## 4. Materials and Methods

### 4.1. Cell Lines, Antibodies, Recombinant Proteins

Raji, RPMI8226, H929, MM1S, K562, CHO, MCF-7, MDA-231, and Lovo cell lines were purchased from the ATCC (Manassas, VA, USA) and cultured either in DMEM (GE Healthcare, Chicago, IL, USA) or in RPMI-1640 medium (ThermoFisher, Waltham, MA, USA) containing 10% FBS (AmCell, Mountain View, CA, USA). CHO-CD37 cells were purchased from BPS Bioscience (San Diego, CA, USA) and cultured in Ham’s F12K medium containing 10% Fetal Bovine Serum, FBS and 1 mg/mL geneticin (ThermoFisher). Hela-CD37 were generated by transducing Hela cells with CD37 lentivirus. Human peripheral blood mononuclear cells (PBMC) from whole blood obtained in the Stanford Hospital Blood Center, Stanford, according to IRB-approved protocol (#13942), were isolated by density sedimentation over Ficoll-Paque (GE Healthcare, San Ramon, CA, USA).

CD37 antibody clone 2B8D12F2D4 was from Promab, (Richmond, CA, USA). Control monoclonal CD37 antibody was from Biolegend (San Diego, CA, USA). Recombinant proteins CD37, CD318, GATA3, CD89, CD43, SP10, MSH2, SERPINA1 were obtained from Promab (Richmond, CA, USA). For ELISA with CD37 and other proteins, HRP labeled anti-Mouse IgG was used from Sigma-Aldrich (St Louis, MO, USA) (Cat#: A0168). Human serum and goat anti-mouse (Fab)2 or anti-human (Fab)2, CD3 antibodies for FACS were from Jackson Immunoresearch (West Grove, PA, USA).

### 4.2. Generation of CD37 Antibody, Clone 2B8D12F2D4

Six-eight weeks old BALB/c mice were immunized by subcutaneous injection, with the recombinant fusion CD37 extracellular domain (109–242 amino-acids of isoform 1 (P11049-1) with C-terminal MBP (Maltose binding protein) and 6× His (histidine) tags. For hybridoma generation, the immunized mice splenocytes were fused with SP2/0 myeloma cells using PEG (Polyethylene glycole) and then hypoxantine (HAT) medium selection. Hybridomas were diluted to obtain single clones on 96-well plates and then were screened by ELISA for the selection of positive clones against the immunogen and unrelated tagged proteins. Several positive hybridoma clones were further cultured and expanded to produce anti-CD37 antibodies. The supernatants of these antibody clones were collected, purified through the Protein G affinity capture column, and analyzed by ELISA, Western, and FACS. The positive clone 2B8D12F2D4 (called 2B8) was selected for VH and VL sequencing and CAR generation.

### 4.3. CAR Lentiviral Construct Design

The codon-optimized sequence ScFv based on CD37, clone 2B8D12F2D4 VH and VL was synthesized in Integrated DNA Technologies (IDT) (San Diego, CA, USA) as a Gblock and subcloned into second-generation CAR sequence with either CD28 costimulatory domain for mouse CD37-CAR-T cells or 4-1BB costimulatory domain for humanized CD37 and bispecific hCD37-CD19 CAR-T cells and CD3 zeta activation domains. The CAR was subcloned into 3^d^ generation lentivirus under either EF1 (with CD28 costimulatory domain CAR) or MNDU3 promoter (with 41BB costimulatory domain CAR). Mock CAR-T cells without ScFv (TF tagged)-CD28-CD3 CAR-T cells were used as Mock CAR-T cells [10].

### 4.4. Humanization of CD37

Humanization of CD37 VH and VL was performed as described before [12,45] by grafting mouse complementarity-determining regions (CDRs) with humanized framework sequences [46].

### 4.5. CAR Lentivirus

2.5 × 10^7^ HEK293FT cells (Thermo Fisher) were seeded on 0.01% gelatin-coated 15 cm plates and cultured overnight in DMEM, 2% FBS, 1xpen/strep, and then transfected with the pPACKH1 Lentivector Packaging mix (System Biosciences, Palo Alto, CA, USA) and 10 μg of the lentiviral vector using the NanoFect transfection reagent NF100 (Alstem, Richmond, CA, USA). The next day the medium was replaced with fresh medium, and 48 h later, the lentivirus-containing medium was collected. The medium was cleared of cell debris by centrifugation at 2100× *g* for 30 min. The virus particles were collected by centrifugation at 112,000× *g* for 60 min at 4 °C using a SW28.1 rotor, suspended in serum-free DMEM medium, aliquoted, and frozen at −80 °C.

### 4.6. CAR-T Cells

PBMC were suspended at 1 × 10^6^ cells/mL in AIM V-AlbuMAX medium (ThermoFisher) containing 10% FBS and 10 ng/mL IL-2 (ThermoFisher), mixed with an equal number (1:1 ratio) of CD3/CD28 Dynabeads (ThermoFisher), and cultured in non-treated 24-well plates (0.5 mL per well). At 24 and 48 h, lentivirus was added to the cultures. The T cells proliferated over the next 10–12 days, the cells were counted every 2–3 days, and fresh medium with 10 ng/mL IL-2 was added to the cultures to maintain the cell density at 1–2 × 10^6^ cells/mL.

### 4.7. Flow Cytometry (FACS)

To measure CAR expression, 0.25 million cells were suspended in 100 μL of buffer (PBS (phosphate buffered saline) containing 2 mM EDTA pH 8 and 0.5% BSA) and incubated on ice with 1 μL of human serum for 10 min. Diluted primary antibody biotin-conjugated goat anti-mouse (Fab)2 or anti-human (Fab)2 was used with cells for 30 min at 4 °C, and after washing, the secondary antibody was added with APC-conjugated mouse α-human CD3 antibody and PE-conjugated streptavidin at 1:100 dilution for 30 min incubation at 4 °C. The cells were rinsed with 3 mL of washing buffer, then stained for 10 min with 7-AAD, suspended in the buffer, and acquired on a FACSCalibur (BD Biosciences, San Jose, CA, USA). Cells were analyzed first for light scatter versus 7-AAD staining, then the 7-AAD^–^ live gated cells were plotted for anti-CD3 staining versus CAR^+^ staining with anti-(Fab)2 antibodies.

### 4.8. Cytotoxicity (RTCA)

Adherent target cells (CHO-CD37; CHO; Hela-CD37 or Hela) were seeded into 96-well E-plates (Acea Biosciences, San Diego, CA, USA) at 1 × 10^4^ cells per well and monitored in culture overnight with the impedance-based real-time cell analysis (RTCA) xCELLigence system (Acea Biosciences). The next day, the medium was removed and replaced with AIM V-AlbuMAX medium containing 10% FBS ± 1 × 10^5^ effector cells (CAR-T cells or non-transduced T cells) in triplicate. The cells in the E-plates were monitored for another 24–48 h with the RTCA system, and impedance was plotted over time. Cytotoxicity was calculated as (impedance of target cells without effector cells—impedance of target cells with effector cells) × 100/impedance of target cells without effector cells.

### 4.9. Affinity Measurement Using SPR Biacore Assay

Anti-CD37 antibody 2B8 clone affinity for CD37 antigen was measured in duplicate experiments on Biacore 3000 using an anti-mouse IgG coated CM5 chip for anti-CD37 antibody 2B8 capture and an activated reference surface. Following Anti-CD37 antibody 2B8 capture in degassed pH 7.4 HEPES (4-(2-hydroxyethyl)-1-piperazineethanesulfonic acid) buffered saline with 0.005% (*w*/*v*) Tween-20 and 3 min stabilization, duplicate serial dilutions of extracellular domain of CD37, CD37-His protein (Prospec, Rehovot, Israel) were injected for 3 min association phase kinetics followed by 15 min dissociation and surface regeneration with pH 1.7 10 mM glycine. The average KD was detected in nM.

### 4.10. ELISA for Detection IFN-Gamma

Nonadherent target cells (Raji, MM1S, K562) were cultured with the effector cells (CAR-T cells or non-transduced T cells) at a 1:1 ratio (1 × 10^4^ cells each) in U-bottom 96-well plates with 200 μL of AIM V-AlbuMAX medium containing 10% FBS, in triplicate. After 16 h, the top 150 μL of the medium was transferred to V-bottom 96-well plates and centrifuged at 300 g for 5 min to pellet any residual cells. The top 120 μL of supernatant was transferred to a new 96-well plate and analyzed by ELISA for human IFN-γ levels using a kit from R&D Systems (Minneapolis, MN, USA) according to the manufacturer’s protocol. The supernatant after RTCA with adherent target cells was collected and analyzed as above.

### 4.11. Mouse Tumor Xenograft Model and Imaging

Six-week-old male NSG mice *(Jackson Laboratories*, Bar Harbor, ME, USA) were housed in accordance with the Institutional Animal Care and Use Committee (IACUC) (# LUM-001). Each mouse was injected subcutaneously on day 0 with 100 μL of 5 × 10^5^ Raji-luciferase positive cells in sterile serum-free medium. The next day 1 × 10^7^ CAR-T cells in serum-free medium were injected intravenously. Imaging was done using Raji-luciferase positive cells after luciferin injection with Xenogen Ivis System. Quantification was done by measuring photons/sec signals. A Kaplan–Myer survival curve was done based on mice survival data.

### 4.12. Immunohistochemistry (IHC)

Tumor tissue sections (4 μm) were deparaffinized in xylenes twice for 10 min, then hydrated in graded alcohols and rinsed in PBS. Antigen retrieval was performed for 20 min in a pressure cooker using 10 mM citrate buffer, pH 6.0. The sections were cooled, rinsed with PBS, incubated in a 3% H_2_O_2_ solution for 10 min, and rinsed with PBS. The tissue sections were incubated in goat serum for 20 min and then incubated with primary CD37 antibody. Then sections were rinsed with PBS, incubated with biotin-conjugated goat anti-mouse IgG for 10 min, rinsed with PBS, incubated with streptavidin-conjugated peroxidase for 10 min, and rinsed with PBS. Finally, the sections were incubated in DAB (3,3’Diaminobenzidine) substrate solution for 2–5 min, immersed in tap water, counterstained with hematoxylin, rinsed with water, and dehydrated in graded alcohols and xylenes. Coverslips were mounted with glycerin. Images were acquired on a Motic DMB5-2231PL microscope with Images Plus 2.0 software (Motic, Xiamen, China).

### 4.13. Statistical Analysis

Data were analyzed and plotted with Prism software (GraphPad V7, San Diego, CA, USA). Comparisons between two groups were performed by unpaired Student’s *t*-test; one or two-way ANOVA, followed by Sidak or Dunnett’s tests for multiple comparisons. The *p*-value < 0.05 was considered significant.

## 5. Conclusions

This study demonstrates that novel CD37, humanized CD37, and humanized CD37-CD19 CAR-T cells specifically targeted CD19 and CD37 positive cells and provides the basis for future clinical studies.

## 6. Patents

The patent application was filed based on the work reported in this manuscript.

## Data Availability

Data is contained within the article or Supplementary Materials.

## Supplementary Materials

The following are available online at https://www.mdpi.com/2072-6694/13/5/981/s1, Figure S1: FACS with CD37 antibody (clone 2B8) shows positive staining in three patient-derived leukemia samples. Figure S2: Mouse and humanized CD37 CAR-T cells significantly block Raji xenograft tumor growth in vivo. Table S1: IHC staining with CD37 antibody (clone 2B8) in different normal and tumor tissues.
