# Supplementary material for: Novel CD37, Humanized CD37 and Bi-Specific Humanized CD37-CD19 CAR-T Cells Specifically Target Lymphoma
Source: Cancers (Basel). 2021 Feb 26;13(5):981. doi: 10.3390/cancers13050981 (PMC7956426; doi:10.3390/cancers13050981)
Supplement: Supplementary file 1 [file cancers-13-00981-s001.pdf]

Supplemental Table 1.

IHC staining with CD37 antibody (clone 2B8) in different normal and tumor tissues

Normal tissue	IHC staining	Tumor tissue	IHC staining
Cardiac muscle	-	Bladder Cancer	-
Muscle	-	Cervical Cancer	-
Cerebellum	-	Endometrial Carcinoma	-
Medulla oblongata	-	Ovarian Cancer	-
Brain	-	Breast Cancer	-
Lung	-	Lung Cancer	-
Tonsil	+	Prostate Carcinoma	-
Pancreas	-/+	Renal Carcinoma	-
Kidney	-	Liver Cancer	-
Liver	-	Rectal Cancer	-
Rectum	-	Colon Cancer	-
Testicle	-	Gastric Cancer	-
Duodenum	-/+	Esophagial Carcinoma	-
Thyroid	-	Cerebroma	+
Stomach	-		
Esophagus	-		

Several normal and tumor tissues were analyzed in two separate fields. Each tissue was analyzed in two different fields in two separate experiments. - shows negative staining; + shows positive staining; -/+ shows heterogenous staining.

Supplemental Figure 1S

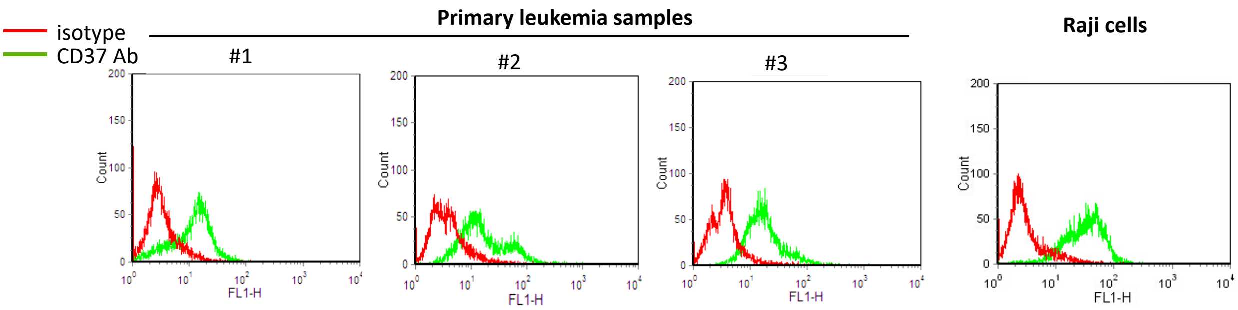

Figure 1S. FACS with CD37 antibody (2B8 clone) shows positive staining in three primary leukemia samples.

Supplemental Figure S2

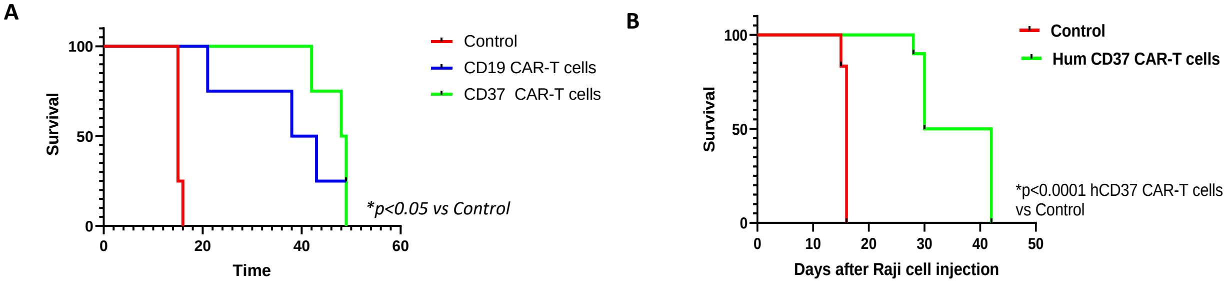

Figure S2. Mouse and Humanized CD37 CAR-T cells significantly block Raji xenograft tumor growth *in vivo. A.* Mouse CD37 CAR-T and CD19 CAR-T cells significantly prolong mouse survival in Raji xenograft mouse model. $p<0.05$, Kaplan-Myer survival vs negative vehicle control group. B. Humanized CD37 CAR-T cells significantly prolong mouse survival in Raji xenograft mouse model. $p<0.0001$, Kaplan-Myer survival vs negative control group
